# B-Cell-Activating Factor Depletion Ameliorates Aging-Dependent Insulin Resistance via Enhancement of Thermogenesis in Adipose Tissues

**DOI:** 10.3390/ijms21145121

**Published:** 2020-07-20

**Authors:** Bobae Kim, Chang-Kee Hyun

**Affiliations:** 1 School of Life Science, Handong Global University, Pohang, Gyungbuk 37554, Korea; bbkim@microbes.bio; 2 HEM Inc., Pohang, Gyungbuk 37554, Korea

**Keywords:** B-cell-activating factor (BAFF), aging-dependent insulin resistance, non-shivering thermogenesis, brown adipose tissue, adipose tissue browning

## Abstract

Impaired glucose tolerance is a common feature associated with human aging, which is caused by defects in insulin secretion, insulin action or both. Recent studies have suggested that B-cell-activating factor (BAFF), a cytokine that modulates proliferation and differentiation of B cells, and its receptors are expressed in mature adipocytes and preadipocytes, proposing BAFF as a potential regulator of energy metabolism. In this study, we show that systemic BAFF depletion improves aging-dependent insulin resistance. In aged (10-month-old) BAFF^−/−^ mice, glucose tolerance and insulin sensitivity were significantly improved despite higher adiposity as a result of expansion of adipose tissues compared to wild-type controls. BAFF^−/−^ mice displayed an improved response to acute cold challenge, commensurate with the up-regulated expression of thermogenic genes in both brown and subcutaneous adipose tissues. These changes were found to be mediated by both increased M2-like (alternative) macrophage activation and enhanced leptin and FGF21 production, which may account for the improving effect of BAFF depletion on insulin resistance. In addition, leptin-deficient mice (ob/ob) showed augmented BAFF signaling concomitant with impaired thermogenic activity, identifying BAFF as a suppressive factor to thermogenesis. Our findings suggest that suppression of BAFF could be a therapeutic approach to attenuate aging-dependent insulin resistance.

## 1. Introduction

B-cell-activating factor (BAFF), a member of the tumor necrosis factor (TNF) ligand family, is a cytokine that plays an important role in the proliferation and differentiation of B cells, which has been shown to be a ligand for receptors: BAFF receptor (BAFF-R), B-cell maturation antigen (BCMA) and transmembrane activator and calcium-modulating cyclophilin ligand interactor (TACI) [1]. Although BAFF was originally identified as a secretory protein in immunocytes, recent studies have described that mature adipocytes also produce BAFF and its receptors, suggesting a role of BAFF in the regulation of energy metabolism [2]. It has recently been reported that a circulating BAFF level is positively correlated with body mass index in individuals with obesity [3], and patients with non-alcoholic fatty liver disease have increased levels of serum BAFF [4]. Several studies using mouse models also have shown a positive correlation between BAFF and energy metabolism dysregulation. Mice chronically fed a high-fat diet had significant increases in the levels of BAFF in serum and visceral adipose tissue [5], and BAFF-R knockout mice were protected from diet-induced adiposity and insulin resistance [6]. 3T3-L1 adipocytes treated with recombinant BAFF protein showed increased expression of resistin and proinflammatory cytokines, decreased production of adiponectin and impaired insulin-mediated glucose uptake [5]. It has also recently been reported that obesity-associated insulin resistance and hepatic steatosis were ameliorated in BAFF-deficient mice fed a high-fat diet [7].

Brown adipose tissue (BAT) is a thermogenic organ that dissipates energy as heat and is characterized by multilocular lipid droplets, high mitochondrial contents and expression of uncoupling protein 1 (UCP1) [8]. Located in the inner mitochondrial membrane of brown adipocytes, UCP1 is essential for adaptive adrenergic non-shivering thermogenesis, which is primarily regulated by the sympathetic nervous system [9]. Catecholamines activate β-adrenergic receptors on BAT, leading to an increase in cAMP-dependent protein kinase activation, thereby increasing the expression of UCP1 and peroxisome proliferator-activated receptor (PPAR)-γ coactivator 1α (PGC1α) among other transcripts relevant for adaptive thermogenesis [10]. UCP1 dissipates the proton gradient generated by the electron transport chain, which drives the oxidation of energy substrates and heat production. Thermogenic capacity of white adipose tissue (WAT) could be augmented by recruitment of beige adipocytes, which exhibit the same cell morphology and UCP1-dependent thermogenic capacity as classic brown adipocytes [11]. The recruitment of beige cells, referred to as “browning”, could be initiated by various stimuli, such as cold exposure, exercise and PPARγ agonists treatment [12,13]. It also has been found that chronic cold exposure can induce eosinophil IL-4/13 production, leading to a recruitment of alternatively activated (M2) macrophages to subcutaneous adipose tissue (SAT) and secrete catecholamines to activate adipose tissue browning [14].

Aging is a degenerative process which is accompanied with functional deterioration in the maintenance of homeostasis [15]. The aging process in energy metabolism is characterized by insulin resistance, expansion and redistribution of WATs, impaired endocrine function and progressive atrophy of BAT [16], which contributes to the development of type 2 diabetes, cardiovascular disease and stroke. In this study, we investigated the impact of BAFF deficiency on aging-dependent insulin resistance using systemic BAFF knockout (BAFF^−/−^) mice. Ten-month-old BAFF^−/−^ mice showed significantly improved insulin sensitivity despite increased body-weight gain, which was attributed to the elevated thermogenesis and augmented gene expression of M2-like macrophage markers and anti-inflammatory cytokines in subcutaneous and brown adipose tissues. Moreover, BAFF^−/−^ mice showed enhanced leptin and FGF21 production, which provides another explanation for the improving effect of BAFF depletion on insulin resistance. On the other hand, leptin-deficient (ob/ob) mice exhibited increased BAFF signaling in both SAT and BAT, with significantly impaired expression of UCP1 in BAT. These results demonstrate that BAFF has an attenuating effect on thermogenic capacity and, thereby, BAFF suppression could improve aging-induced insulin resistance by promoting non-shivering thermogenesis.

## 2. Results

### 2.1. Protective Effect of BAFF Deficiency against Aging-Dependent Insulin Resistance

**Figure 1:**
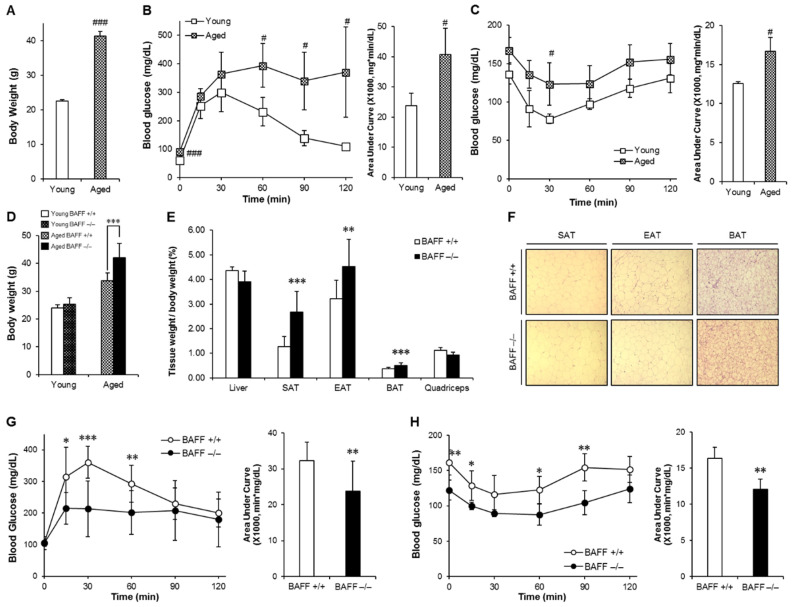
Aging aggravates insulin resistance and aged BAFF^−/−^ (B-cell-activating factor) mice shows enhanced glucose tolerance despite increased body weight. Young and old mice were maintained on a normal chow diet for 2 months and 10 months, respectively. Changes of (**A**) body weight, (**B**) glucose tolerance and (**C**) insulin sensitivity were measured (*n* = 5). Mice fasted for 16 h, and the blood glucose levels were measured at 0, 15, 30, 60, 90 and 120 min after intraperitoneal injection of glucose (2 g/kg) or insulin (0.5 U/kg). BAFF^−/−^ and C57BL/6J mice were maintained on a normal chow diet for 10 months. (**D**) Body weights of 2- and 10-month-old adult male mice (*n* = 4–12). (**E**) Tissue weights of 10-month-old mice (*n* = 9–12). (**F**) Hematoxylin and eosin (H&E) staining sections of adipose tissues. Adipose tissues were fixed in 10% *v/v* formalin/PBS, and then embedded in paraffin for staining with hematoxylin and eosin. Images were obtained under a microscope at a magnification of X100. (**G** and **H**) Glucose tolerance test and insulin tolerance test of 10-month-old male mice (*n* = 8–12). Data represent means ± SD. # *p* < 0.05 and ### *p* < 0.001 between young and old mice and * *p* < 0.05, ** *p* < 0.01, *** *p* < 0.001 between wild-type and BAFF^−/−^ mice. SAT: subcutaneous adipose tissue, EAT: epididymal adipose tissue, BAT: interscapular brown adipose tissue.

We first examined the consequences of aging on body weight and glucose control in wild-type (WT) mice. Aged (10-month-old) mice showed significantly increased body weight accompanied with impaired glucose and insulin tolerance when compared to young (2-month-old) counterparts, indicating that aging is associated with insulin resistance (Figure 1A–C). Next, to examine the effects of BAFF depletion on aging-induced insulin resistance, we compared glucose tolerance and insulin sensitivity between aged BAFF^−/−^ and WT mice. As shown in Figure 1D, aged BAFF^−/−^ mice showed significantly higher body weight than WT controls, which was associated with commensurate increases in weight of adipose tissues, including SAT, EAT (epididymal adipose tissue) and BAT (Figure 1E). There were no differences in food intake (Figure S1) and adipocyte hypertrophy (Figure 1F and Figure S2) between the two groups. Interestingly, despite increased adiposity, BAFF^−/−^ mice exhibited significantly enhanced glucose tolerance and insulin sensitivity compared to their WT controls (Figure 1G,H), indicating that knockout of BAFF resulted in improved insulin resistance in aged mice.

### 2.2. Enhancing Effect of BAFF Deficiency on Thermogenesis in BAT and SAT

**Figure 2:**
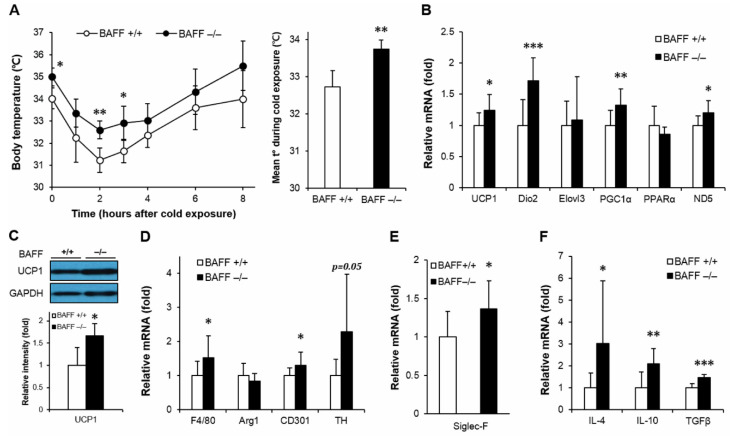
BAFF depletion supports uncoupling protein 1 (UCP1)-dependent thermogenesis and alternative macrophage activation in brown adipose tissue. (**A**) Body core temperature of 10-month-old wild-type or BAFF^−/−^ mice. Measurements were performed at 0, 1, 2, 3, 4, 6 and 8 h during cold exposure at 4 °C (*n* = 4–5). (**B**) Effect of BAFF deficiency on thermogenic program gene expression (*n* = 9–12). Gene expression level is normalized with mRNA expression level of Arbp. (**C**) Effect of BAFF deficiency on UCP1 protein expression level (*n* = 4–5). Proteins were extracted from the tissue for SDS-PAGE-immunoblot analysis. Effect of BAFF deficiency on the expression of genes involved in (**D**) M2-like macrophage activation, (**E**) mature eosinophil cell marker and (**F**) anti-inflammatory cytokines (*n* = 9–12). Data represent means ± SD. * *p* < 0.05, ** *p* < 0.01, *** *p* < 0.001 between wild-type and BAFF^−/−^ mice.

**Figure 3:**
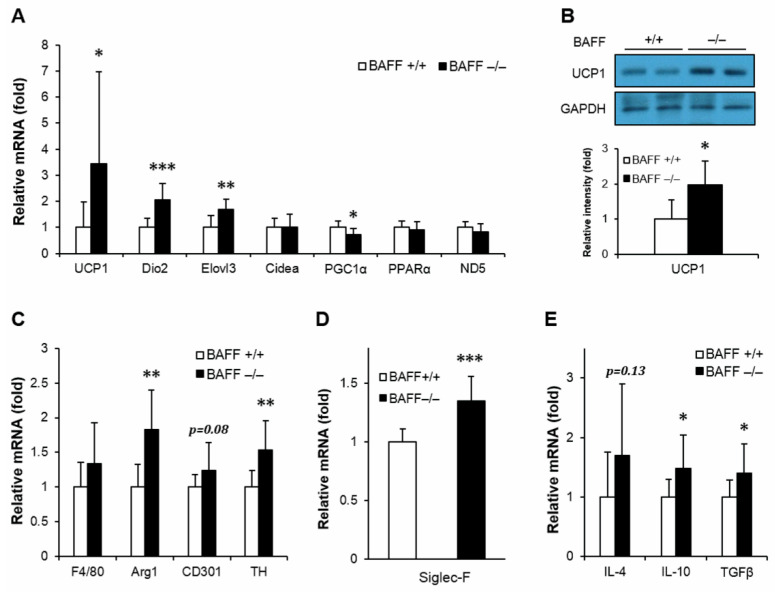
BAFF depletion enhances expression of genes involved in thermogenic program and alternative macrophage activation in subcutaneous adipose tissue. (**A**) Effect of BAFF deficiency on thermogenic program gene expression (*n* = 9–12). Gene expression level is normalized with mRNA expression level of Arbp. (**B**) Effect of BAFF deficiency on UCP1 protein expression level (*n* = 4–5). Proteins were extracted from the tissue for SDS-PAGE-immunoblot analysis. Effect of BAFF deficiency on the expression of genes involved in (**C**) M2-like macrophage activation, (**D**) mature eosinophil cell marker and (**E**) anti-inflammatory cytokine (*n* = 9–12). Data represent means ± SD. * *p* < 0.05, ** *p* < 0.01, *** *p* < 0.001 between wild-type and BAFF^−/−^ mice.

It has been known that thermogenic activity declines during aging, which contributes to the development of metabolic disorders [16]. It has also been demonstrated that activation of thermogenic program exerts protective effects against insulin resistance [13]. To examine whether BAFF deficiency modulates thermogenic capacity, aged BAFF^−/−^ and WT mice were exposed to cold (4 °C) and followed by measurement of rectal temperature with exposure time. In response to the cold challenge, BAFF^−/−^ mice displayed a strong resistance to acute temperature drop compared to their WT counterparts (Figure 2A). We also observed that the skin temperature of BAFF^−/−^ mice pups at day 8 after birth was higher than that of WT controls (Figure S2). Analysis of gene expression in BAT revealed that BAFF deficiency significantly increased mRNA expression of genes involved in thermogenesis such as UCP1, Dio2, PGC1α and mitochondrial gene ND5 (Figure 2B). Expression of UCP1 protein was also substantially higher in BAFF^−/−^ than that in WT mice (Figure 2C). Additionally, the augmentation of expression of thermogenic genes and UCP1 protein was also observed in a beige adipose tissue depot (SAT) of BAFF^−/−^ mice when compared to their WT control mice (Figure 3A,B).

Adipose tissue macrophage is one of the key mediators of insulin resistance [17]. Alteration of monocyte infiltration and differentiation toward a classically (M1) or alternatively (M2) activated macrophage modulates inflammatory and thermogenic microenvironment of adipocytes, which directly contributes to the development of insulin resistance. We observed an increasing tendency in mRNA expressions of pan-macrophage marker, F4/80, and alternatively activated macrophage markers, arginase 1 (Arg1) and CD301, in both BAT and SAT of BAFF^−/−^ mice compared to those of WT mice (Figure 2D and Figure 3C). In addition, expression levels of tyrosine hydroxylase (TH), the rate-limiting enzyme in catecholamine biosynthesis; Siglec-F, a mature eosinophil-specific marker; and anti-inflammatory cytokines, such as IL-4, IL-10 and TGFβ, were significantly increased in BAFF^−/−^ mice (Figure 2D–F and Figure 3C–E). Taken together, these observations suggest that BAFF deficiency augments thermogenic capacity, which is accompanied with the increase of anti-inflammatory response mediated by M2-like macrophages and eosinophils in BAT and SAT.

### 2.3. Effect of BAFF Deficiency on Leptin and FGF21 Production

**Figure 4:**
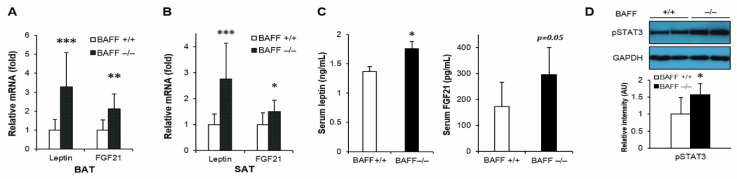
BAFF depletion enhances expression of leptin and FGF21 in subcutaneous and brown adipose tissues. Effect of BAFF deficiency on leptin and FGF21 mRNA expression in (**A**) BAT and (**B**) SAT (*n* = 9–12). Gene expression level is normalized with mRNA expression level of Arbp. (**C**) Effect of BAFF deficiency on serum protein levels of leptin and FGF21 (*n* = 6–8). (**D**) Effect of BAFF deficiency on leptin-dependent STAT3 phosphorylation in brown adipose tissue of 10-month-old mice. Proteins were extracted from BAT for SDS-PAGE-immunoblot analysis (*n* = 4–5). Data represent means ± SD. * *p* < 0.05, ** *p* < 0.01, *** *p* < 0.001 between wild-type and BAFF^−/−^ mice.

We have previously reported that BAFF^−/−^ mice fed with a high-fat diet had enhanced leptin and FGF21 expression in adipose tissues [18]. Moreover, in this study it was also found that the mRNA expressions of leptin and FGF21 were significantly up-regulated in both BAT and SAT of aged BAFF^−/−^ mice compared to their WT controls (Figure 4A,B). Consistent with the increased mRNA levels, serum concentrations of leptin and FGF21 were also considerably higher in BAFF^−/−^ mice than WT controls (Figure 4C). In addition, phosphorylation of STAT3, which mediates leptin action [19], was significantly higher in BAT of BAFF^−/−^ mice than in WT counterparts (Figure 4D).

### 2.4. Tissue-Specific Up-Regulation of BAFF Production and BAFF Signaling in ob/ob Mice

**Figure 5:**
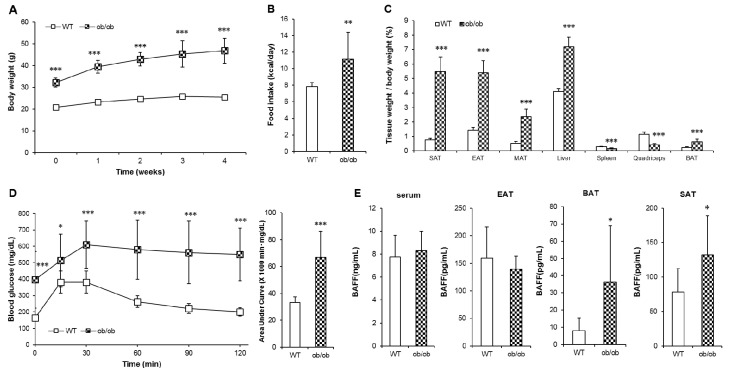
ob/ob mice shows deteriorated glucose tolerance with tissue-specific increase of BAFF expression. Leptin-deficient (ob/ob) mice and C57BL/6J (wild-type, WT) mice were maintained on a normal chow diet for 5 weeks. (**A**) Changes of body weight over 5 weeks (*n* = 10). Changes of (**B**) average calorie intake and (**C**) tissue weights (*n* = 10). (**D**) Glucose tolerance test of WT and ob/ob mice (*n* = 10). Mice fasted for 16 h, and the blood glucose levels were measured at 0, 15, 30, 60, 90 and 120 min after intraperitoneal injection of glucose (2 g/kg). (E) Protein BAFF concentration in serum and adipose tissues (*n* = 8). Data represent means ± SD. * *p* < 0.05, ** *p* < 0.01, *** *p* < 0.001 between wild-type and ob/ob mice. SAT: subcutaneous adipose tissue, EAT: epididymal adipose tissue, MAT: mesenteric adipose tissue, BAT: interscapular brown adipose tissue.

Mice with homozygous mutation in leptin gene (ob/ob) exhibit not only obesity-induced insulin resistance but also hypothermia [20]. To examine if BAFF would affect any part of hypothermia exhibited by the ob/ob mice, we analyzed the tissue-specific expression of BAFF in ob/ob mice. Consistent with known physiological characteristics, ob/ob mice showed significantly higher body weight and calorie intake than those of WT mice (Figure 5A,B) in parallel with dramatic increases in weight of tissues including SAT, EAT, MAT (mesenteric adipose tissue), BAT and the liver (Figure 5C). The onset of hyperglycemia and glucose intolerance was also observed in ob/ob mice (Figure 5D). Unexpectedly, despite markedly increased adiposity, the serum BAFF concentration of ob/ob mice was not different from that of their WT controls (Figure 5E). Interestingly, however, the expression of BAFF protein was significantly higher in SAT and BAT, but not in EAT, of ob/ob mice than in WT controls. This indicates that, under leptin-deficient conditions, BAFF is up-regulated in a tissue-specific manner, i.e. only in thermogenic adipose tissues.

**Figure 6:**
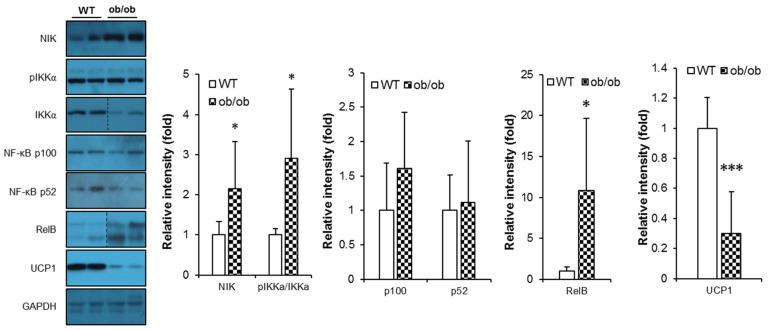
BAFF signaling is up-regulated in brown and subcutaneous adipose tissues of ob/ob mice. Effect of leptin deficiency on protein levels of BAFF signaling molecules and UCP1 in brown adipose tissue (*n* = 6–8). Proteins were extracted from the tissue for SDS-PAGE-immunoblot analysis. Data represent means ± SD. * *p* < 0.05 and *** *p* < 0.001 between wild-type and ob/ob mice.

We next examined signaling molecules connecting BAFF-R to downstream non-canonical NF-κB activation pathway [1]. It was observed that ob/ob mice had significantly up-regulated expression of signaling components of BAFF signaling pathway in BAT compared to their WT controls (Figure 6), while this up-regulation was not clearly shown in SAT (Figure S5). In addition, the protein level of UCP1 was also significantly decreased in BAT of ob/ob mice (Figure 6), indicating that reduced thermogenesis was associated with promoted action of BAFF under leptin deficiency.

## 3. Discussion

Several studies have reported positive correlations between plasma BAFF level and metabolic diseases, such as insulin resistance and non-alcoholic fatty liver disease [3,4,5,6,7]. We also previously reported a protective effect of BAFF depletion against high-fat diet-induced glucose intolerance using BAFF^−/−^ mice on a high-fat diet, which exhibited improved insulin sensitivity and potentiated adipose tissue function [18]. These studies have provided insights into the mechanisms of how the suppression of BAFF activity improves energy metabolism, which include suppression of proinflammatory responses and promotion of BAT activity.

Aging and metabolic syndrome have many predisposing conditions in common, such as obesity, insulin resistance, chronic inflammation, oxidative stress and hypertension [21]. In this study, statistically significant impairment in glucose tolerance and insulin sensitivity was observed in aged WT mice compared with their young controls (Figure 1A–C). We also observed that BAFF depletion triggered an increase of weight gain in aged mice compared with their young controls (Figure 1D). In our previous studies [18,22], we observed that the lipogenic activity was enhanced in adipose tissues of BAFF^−/−^ mice compared to WT controls, which was supported by data showing that BAFF depletion caused an attenuated inhibition of PPARγ activity and an increased expression of lipogenic genes. In this study, with commensurate increase in the weight of adipose tissues, the weight gain of aged BAFF^−/−^ mice was higher than that of WT controls, which was not the case for young BAFF^−/−^ mice (Figure 1D). These data collectively suggest that the impact of BAFF depletion on weight gain is aging-dependent, which is caused by, at least in part, an enhanced lipogenic activity of adipose tissues in BAFF^−/−^ mice. Interestingly, however, despite increased adiposity, BAFF^−/−^ mice showed improved insulin sensitivity (Figure 1G,H), suggesting that BAFF depletion ameliorates aging-dependent insulin resistance. It is known that adipose tissue hyperplasia is significantly associated with improved glucose profile compared with adipose hypertrophy [23]. In our study, we observed no difference in adipocyte size between aged BAFF^−/−^ mice and their WT controls (Figure 1F and Figure S2), while adiposity increased in aged BAFF^−/−^ mice, which indicates that BAFF depletion improves insulin resistance, at least in part, through promoting adipose tissue expansion. The enhanced lipogenesis also might contribute to promoted adipose tissue expansion, and thereby lead to the improved insulin sensitivity in aged BAFF^−/−^ mice. On the other hand, it was observed in our previous studies that BAFF deficiency potentiates adipose tissue function mediated by FGF21 and leptin [18] and enhances BAT thermogenesis [22]. From the light of these findings, we focused on browning and thermogenesis of adipose tissue to examine the impact of BAFF deficiency on insulin resistance in aged mice.

Activation of BAT, a thermogenic tissue which converts lipid into heat, plays a key role in thermoregulation and exerts beneficial effects on adiposity, glucose intolerance and hyperlipidemia [7]. It has been reported that the aging process is associated with an expansion of white, specifically visceral, fat and an involution of BAT, resulting in an increasing propensity to develop obesity and aging-related metabolic disorders [16]. Evidence also indicates that reduced thermogenic capacity in aged animals and humans are due, at least in part, to decreased sensitivity to β-adrenergic stimulation [24]. In the present study, we found that aged BAFF^−/−^ mice had a significant increase in non-shivering thermogenesis in response to an acute cold challenge (Figure 2A). In accordance with this increased thermogenic capacity, genes involved in the thermogenic program were up-regulated in both BAT (Figure 2B,C) and SAT (Figure 3A,B) of BAFF^−/−^ mice. These findings suggest that the insulin sensitizing effect of BAFF depletion is, at least in part, mediated by activation and/or recruitment of brown and beige adipocytes.

The activation and recruitment of thermogenic adipocytes could be facilitated in response to various cues, including β-adrenergic stimulation, non-adrenergic peptide hormones, and thyroid and PPARγ agonists [25,26]. Recent studies have proposed that macrophages recruited to cold-stressed SAT undergo alternative (M2) activation to induce catecholamine production, leading to β-adrenergic stimulation and WAT browning [14]. Exposure to cold rapidly promotes M2 macrophage polarization, which is accompanied by increased expression of genes, such as Arg1, CD206, and CD301 and secretion of anti-inflammatory cytokines, including IL-10 and TGFβ [27]. Then, in the alternatively activated (M2) macrophages, signals triggered by eosinophil-derived IL-4 pathway induces the expression of tyrosine hydroxylase, dopamine decarboxylase and β-hydroxylase, which are responsible for synthesis of catecholamine to sustain adaptive thermogenesis [28]. Given these well-established associations, we hypothesized in our study that BAFF depletion would be positively related to M2 macrophage induction and β-adrenergic stimulation, followed by enhanced browning of WAT. We observed that BAFF^−/−^ mice displayed enhanced M2 macrophage activation and accumulation of mature eosinophil in both BAT (Figure 2D–F) and SAT (Figure 3C–E), suggesting that β-adrenergic stimulation by immunocytes plays a role in the BAFF depletion-induced browning.

Apart from the stimulatory effect on catecholamine synthesized by macrophages, we found that BAFF deficiency also stimulated the production of FGF21 and leptin in adipose tissues. FGF21 is known to mediate the browning and thermogenesis of adipose tissue, which leads to an improvement of insulin resistance and a reduction of ectopic fat accumulation [29]. Leptin is also known to have a function in non-shivering thermogenesis. Several studies have shown that elevated levels of circulating leptin result in the enhancement of glucose utilization by inducing UCP1 expression and STAT phosphorylation in BAT [19,30]. In the present study, the two hormones were significantly up-regulated in BAT and SAT of aged BAFF^−/−^ mice when compared to their WT counterparts, both in mRNA and protein levels (Figure 4). We also observed that STAT3 phosphorylation in BAT of aged BAFF^−/−^ mice was significantly increased compared to WT controls (Figure 4D), indicating an enhanced activity of leptin. These findings suggest that the enhancement of thermogenic capacity and consequent improvement of insulin resistance in BAFF^−/−^ mice was mediated, at least in part, by FGF21 and leptin. In contrast to the contribution of adipose tissue to the improvement of insulin resistance in aged BAFF^−/−^ mice, the liver was not a significant player. In this study, no evidence was found that BAFF depletion caused any significant alterations in blood lipid levels and hepatic expression of genes related to glucose and lipid metabolism (Figure S4). This is inconsistent with the results of a recent study, which reported on the ameliorating effect of BAFF depletion on obesity-associated insulin resistance and hepatic steatosis [7]. This inconsistency may be due to the differences in diet condition and the age of mice between the two studies.

We still had to explore the mechanism for why aged BAFF deficient mice, despite having increased adiposity, exhibited improved glucose tolerance compared to their WT counterparts. From the fact that adipose tissues produce significant amount of BAFF, we hypothesized that the enhancing effect of BAFF depletion on thermogenic capacity would be prominent in the conditions of higher expansion of adipose tissues. To test this hypothesis, the ob/ob mouse was selected as an appropriate model of hypothermia because it is morbidly obese, hypothermic, and diabetic [20]. Consistent with known and expected phenotypes of ob/ob mice, we observed severe obesity, tissue adiposity and glucose intolerance in ob/ob mice (Figure 5A–D), with significantly decreased UCP1 level in BAT (Figure 6). Interestingly, however, despite extreme adiposity, serum BAFF concentration was not altered in ob/ob mice compared to WT controls (Figure 5E), which was distinct from high-fat diet-induced obese mice that exhibited severe obesity and insulin resistance accompanied with a significantly increased serum BAFF level compared to normal diet-fed mice [5]. Moreover, the expression pattern of BAFF protein in ob/ob mice was observed to be tissue-specific. BAFF protein level was significantly higher in SAT and BAT, but not EAT, of ob/ob mice than WT controls (Figure 5E).

It has been known that BAFF preferentially binds to BAFF-R and triggers the non-canonical NF-κB pathway [1,31]. In the absence of BAFF stimulation, TNF receptor-associated factor 3 (TRAF3) binds to NF-κB-inducing kinase (NIK) and induces ubiquitin-dependent proteolysis, thus inhibiting the activation of the non-canonical NF-κB pathway. In contrast, when BAFF binds to BAFF-R, TRAF3 is recruited to BAFF-R and subsequently degrades, resulting in the stabilization of NIK, followed by activation of IκB kinase-α (IKKα) that phosphorylates NF-κB p100. The phosphorylated p100 is then processed to produce NF-κB p52, which creates the NF-κB p52/RelB complex that translocates to the nucleus and induce target gene expression [1,31]. It also has been reported that adipocyte-specific RelB knockout mice showed improved insulin sensitivity despite increased adiposity, which was suggested to be mediated by suppression of the non-canonical NF-κB pathway in adipose tissues [32]. In our study, in accordance with the selective increase of BAFF production in BAT of ob/ob mice, expression and processing of BAFF-R signaling components were also up-regulated (Figure 6). This association provides another explanation for how insulin resistance could be improved in spite of increased adiposity in aged BAFF^−/−^ mice of this study. Although aged BAFF^−/−^ mice had a greatly increased expansion of adipose tissues, resulting in increased body weight compared to their WT controls, their insulin sensitivity was significantly improved, possibly attributed to blockade of non-canonical NF-κB pathway caused by elimination of BAFF action in adipose tissues. In addition, from our observation of increased BAFF expression in SAT and BAT of ob/ob mice, we could deduce that the hypothermia of leptin-deficient mice is, at least in part, attributed to an anti-thermogenic action of BAFF in SAT and BAT. Our observation also demonstrated that augmentation of thermogenesis by BAFF depletion is independent of circulating BAFF proteins; rather, BAFF-dependent modulation of thermogenic activity is a tissue-intrinsic regulation. Taken together, our data obtained from experiments using ob/ob mice suggest that BAFF up-regulation contributes to suppression of non-shivering thermogenic activity and consequent insulin resistance under leptin-depleted condition. However, to conclude a causal association of BAFF suppression with enhanced thermogenesis, further study to prove the direct link between BAFF signaling and thermogenic program is needed.

In summary, we found that BAFF deficiency protects mice against aging-dependent insulin resistance via not only promotion of adipose tissue expansion and lipogenesis, but also augmentation of thermogenic capacity of adipose tissues. Especially for enhanced thermogenesis, BAFF deficiency potentiates adipose tissues through increasing M2-macrophage-mediated catecholamine synthesis and enhancing the production of leptin and FGF21. BAFF up-regulation, concomitant with activated BAFF signaling pathway, exhibited in SAT and BAT of leptin-deficient mice confirms that BAFF acts suppressively to non-shivering thermogenesis and may consequently result in exacerbation of insulin resistance. Conclusively, the possible mechanisms by which BAFF deficiency ameliorates aging-dependent insulin resistance are still not completely clear but include the promotion of hyperplasia, lipogenesis and thermogenic activity in adipose tissues. Our findings identify BAFF as a therapeutic target to improve insulin sensitivity with potential applications in the prevention and treatment of aging-associated glucose metabolic disorders.

## 4. Materials and Methods

### 4.1. Animals

BAFF^−/−^ mice on a C57BL/6J background were generated by insertion of the tailless human CDs reporter gene into the BAFF locus (Bar Harbor, ME; stock number 010572). BAFF gene deletion was confirmed by analysis of mRNA and protein expression of BAFF in spleen, epididymal and brown adipose tissue as described previously [22]. Mice were fed with a normal chow diet (2018S, Harlan Laboratories, Indianapolis, IN, USA) and maintained under a 12 h light/dark cycle at a constant temperature of 22 ± 1 °C and humidity of 45 ± 10%.

Mice fasted for 4 h and were sacrificed by cervical dislocation. Tissues of the liver, spleen, inguinal subcutaneous adipose tissue (SAT), epididymal adipose tissue (EAT), interscapular brown adipose tissue (iBAT) and quadriceps were harvested, snap-frozen in liquid nitrogen, and stored at −70 °C until processed for RNA and protein analysis. All the experimental protocols were approved by the Committee on the Ethics of Animal Experiments of the Handong Global University (Approval No., HGUIACUC20151022-010; Approval date, 22 October 2015).

### 4.2. Core Body Temperature Measurements and Cold-Tolerance Test

Mice were placed in prechilled cages at 4 °C with free access to a normal chow diet and water. Core body temperatures were measured at 0, 1, 2, 3, 4, 6 and 8 h during cold exposure using an electronic rectal thermometer Testo 925 (Testo, Inc., Lenzkirch, Germany).

### 4.3. Glucose Tolerance Test and Insulin Tolerance Test

For the glucose tolerance test, mice fasted for 16 h and then received an intraperitoneal injection of glucose (2 g/kg). For the insulin tolerance test, mice fasted for 4 h and were injected with 0.5 U/kg insulin. Blood samples were obtained by tail-bleeding, and glucose levels were measured at 0, 15, 30, 60, 90 and 120 min after glucose or insulin injection by GlucoDr auto AGM-4000 (Allmedicus, Anyang, Republic of Korea).

### 4.4. Histological Analysis

Adipose tissue samples from each mouse were fixed in 10% *v/v* formalin/PBS, and then embedded in paraffin for staining with hematoxylin and eosin (H&E). Images were obtained under a Carl Zeiss light microscope (Carl Zeiss Microscopy GmbH, Göttingen, Germany) at a magnification of ×100. Adipocyte size was quantified using ImageJ software (National Institutes of Health, Bethesda, MD, USA).

### 4.5. Real-Time RT PCR

Total RNA was extracted using a TRI reagent (Molecular Research Center, Cincinnati, OH, USA) and reverse transcribed with oligo (dT) primer and GoScript^TM^ reverse transcription system (Promega, Madison, WI, USA). Quantitative PCR of gene transcripts for arginase 1 (Arg1), CD301, cell death-inducing DNA fragmentation factor α subunit-like effector A (Cidea), type II iodothyronine deiodinase (Dio2), elongation of very long chain fatty acids protein 3 (Elovl3), epidermal growth factor-like module-containing mucin-like hormone receptor-like 1 (F4/80), fibroblast growth factor 21 (FGF21), interleukin-4 (IL-4), IL-10, leptin, mitochondrially encoded NADH dehydrogenase subunit 5 (ND5), peroxisome proliferator-activated receptor γ coactivator 1α (PGC1α), peroxisome proliferator-activated receptor α (PPARα), sialic acid-binding immunoglobulin-like lectin F (Siglec F), transforming growth factor β (TGFβ), tyrosine hydroxylase (TH) and uncoupling protein 1 (UCP1) were performed by using gene-specific primers. Primer sequences are available upon request. Results were presented as mean ± SD normalized to expression of 36B4 (Arbp) using the ΔΔCt method.

### 4.6. Enzyme-Linked Immunosorbent Assay (ELISA)

Measurements of BAFF, FGF21 (R&D systems, Minneapolis, MN), insulin (Morinaga Institute of Biological Science Inc., Yokohama, Japan) and leptin (Elabscience Biotechnology Inc., Houston, TX, USA) were performed with commercial ELISA kits according to the manufacturer’s instructions.

### 4.7. Western Blotting

Western blotting was performed as described previously [22]. Tissues were homogenized in ice-cold PRO-PREP protein extraction buffer (iNtRON Biotechnology, Seongnam, Republic of Korea), and centrifuged at 16,600 g for 10 min at 4 °C. Supernatants were collected, boiled, and analyzed by an SDS-PAGE-immunoblotting assay. Antibodies against GAPDH, phospho-IKKα/β (Ser176+Ser180), IKKα (Bioss Antibodies, Woburn, MA, USA), NF-κB p100/p52, NIK, RelB (Cell Signaling Technology, Berverly, MA, USA), phospho-STAT3 (Tyr705) (Cambridge Bioscience, Cambridge, UK) and UCP1 (Abcam, Cambridge, UK) were used as primary antibodies, followed by the appropriate IgG-HRP conjugated secondary antibody (Cell Signaling Technology). Proteins were visualized by ECL.

### 4.8. Statistical Analysis

All data were presented as mean ± SD Comparisons between two groups were performed by two-tailed Student’s *t*-test. *p* values <0.05 were considered as statistically significant.

## Supplementary Materials

Supplementary materials can be found at https://www.mdpi.com/1422-0067/21/14/5121/s1. Figure S1. There was no significant difference in food intake between aged BAFF^−/−^ mice and their WT controls. Figure S2. There was no significant difference in adipocyte hypertrophy between aged BAFF^−/−^ mice and their WT controls. Figure S3. Skin body temperature of BAFF^−/−^ and WT mice pups 8 days after birth. Figure S4. There were no significant differences in blood lipid concentration and expression level of genes related to glucose and lipid metabolism between aged BAFF^−/−^ mice and their WT controls. Figure S5. NF-κB signaling in SAT of ob/ob mice.

## Abbreviations

BAFF	B-cell activating factor
BAT	Brown adipose tissue
EAT	Epididymal adipose tissue
SAT	Subcutaneous adipose tissue
UCP1	Uncoupling protein 1
