# Supplementary material for: B-Cell-Activating Factor Depletion Ameliorates Aging-Dependent Insulin Resistance via Enhancement of Thermogenesis in Adipose Tissues
Source: Int J Mol Sci. 2020 Jul 20;21(14):5121. doi: 10.3390/ijms21145121 (PMC7404107; doi:10.3390/ijms21145121)
Supplement: Supplementary file 1 [file ijms-21-05121-s001.pdf]

International Journal of *Molecular Sciences*

Supplementary Information

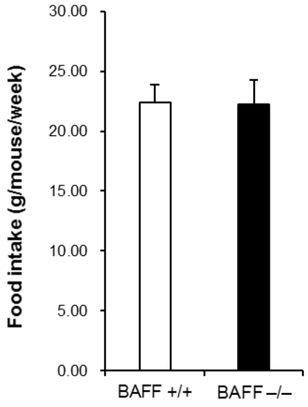

Figure S1. There was no significant difference in food intake between aged BAFF$^{-/-}$ mice and their wild-type controls.

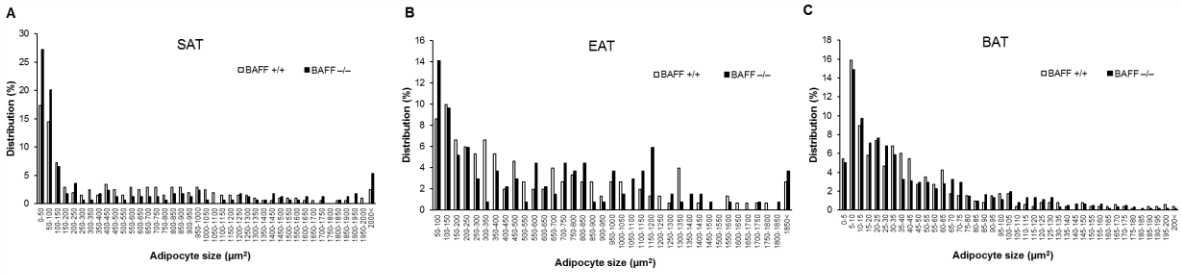

Figure S2. There was no significant difference in adipocyte hypertrophy between aged BAFF$^{-/-}$ mice and their wild-type controls.

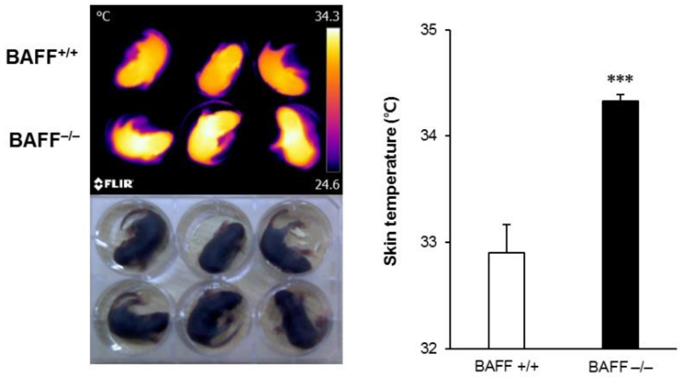

Figure S3. Skin body temperature of BAFF$^{-/-}$ and wild-type mice pups 8 days after birth.

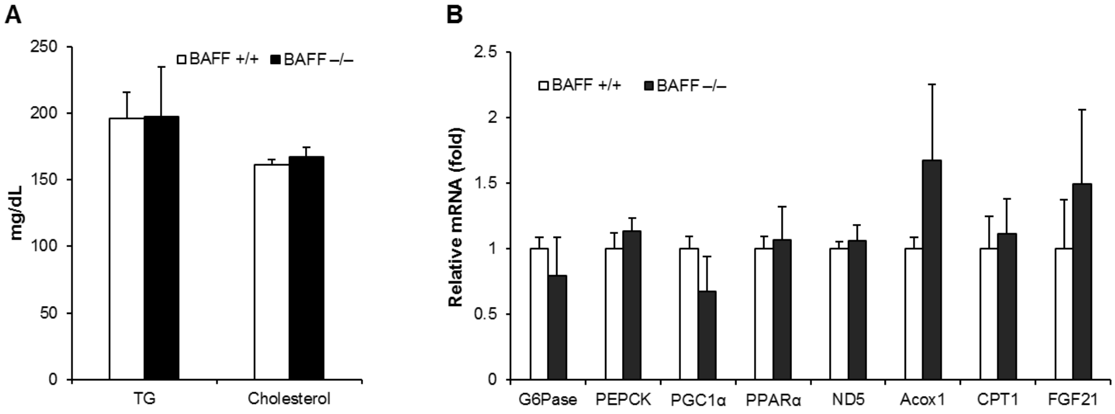

Figure S4. There were no significant differences in blood lipid concentration and expression level of genes related to glucose and lipid metabolism between aged BAFF$^{-/-}$ mice and their wild-type controls.

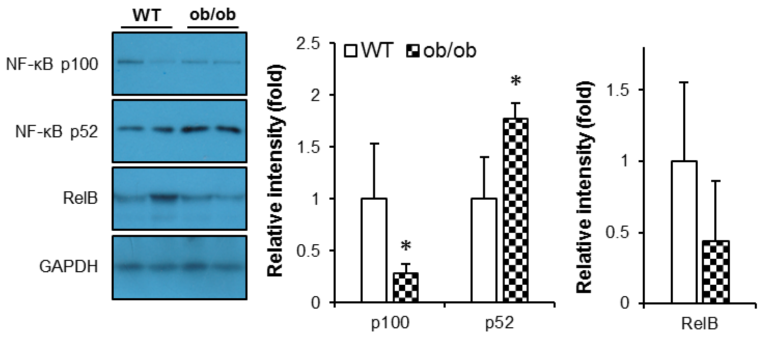

Figure S5. NF-κB signaling in subcutaneous adipose tissue of ob/ob mice.

© 2020 by the authors. Submitted for possible open access publication under the terms and conditions of the Creative Commons Attribution (CC BY) license (http://creativecommons.org/licenses/by/4.0/).
